# Supplementary material for: A naturally selected αβ T cell receptor binds HLA-DQ2 molecules without co-contacting the presented peptide
Source: Nat Commun. 2025 Apr 8;16:3330. doi: 10.1038/s41467-025-58690-w (PMC11979002; doi:10.1038/s41467-025-58690-w)
Supplement: Supplementary file 1 — Supplementary Information [file 41467_2025_58690_MOESM1_ESM.pdf]

Supplementary Information

A naturally selected αβ T cell receptor binds HLA-DQ2 molecules without co-contacting the presented peptide

Jia Jia Lim[1], Claerwen M. Jones[1], Tiing Jen Loh[1], Hien Thy Dao[1], Mai T. Tran[1], Jason A. Tye-Din[2], Nicole L. La Gruta[#1] & Jamie Rossjohn[#1,3]

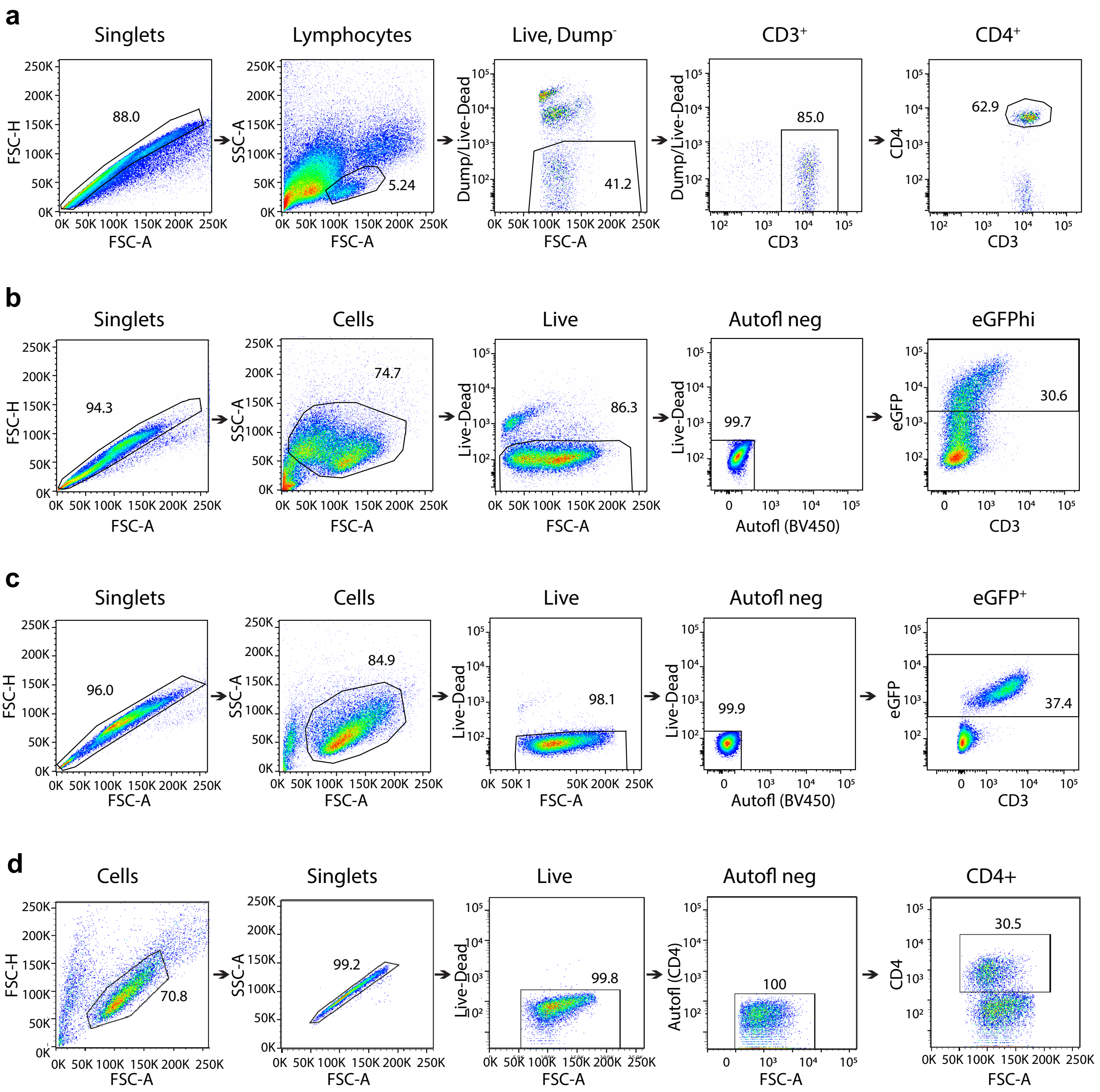

Supplementary Figure 1. Flow cytometry gating strategies. Numbers in black type indicate percentage of cells within a gate. **a** Gating strategy for the detection of HLA-DQ5$^{glia\text{-}\omega1}$ and HLA-DQ5$^{glia\text{-}\omega2}$ CD4^{+} T cells in PBMC isolated from HLA-DQ2.5/DQ2.5 coeliac disease donor, presented in Fig. 1a. **b** Representative gating strategy for the detection of tetramer-binding on 293T cells transiently co-transfected with selected TCR and CD3γδεζ, presented in Fig. 1b and supplementary Fig. 2a. **c-d** Representative gating strategy for the detection of CD69 expression on **(c)** Jurkat cells and **(d)** SKW3 cells transduced with G9- or G2 TCR and stimulated with RAJI B cells coated with glia peptide, presented in Fig. 7a & b. A similar gating strategy was used on TCR-transduced Jurkat cells and SKW3 cells for detection of tetramer-binding presented in supplementary Fig. 9a & b.

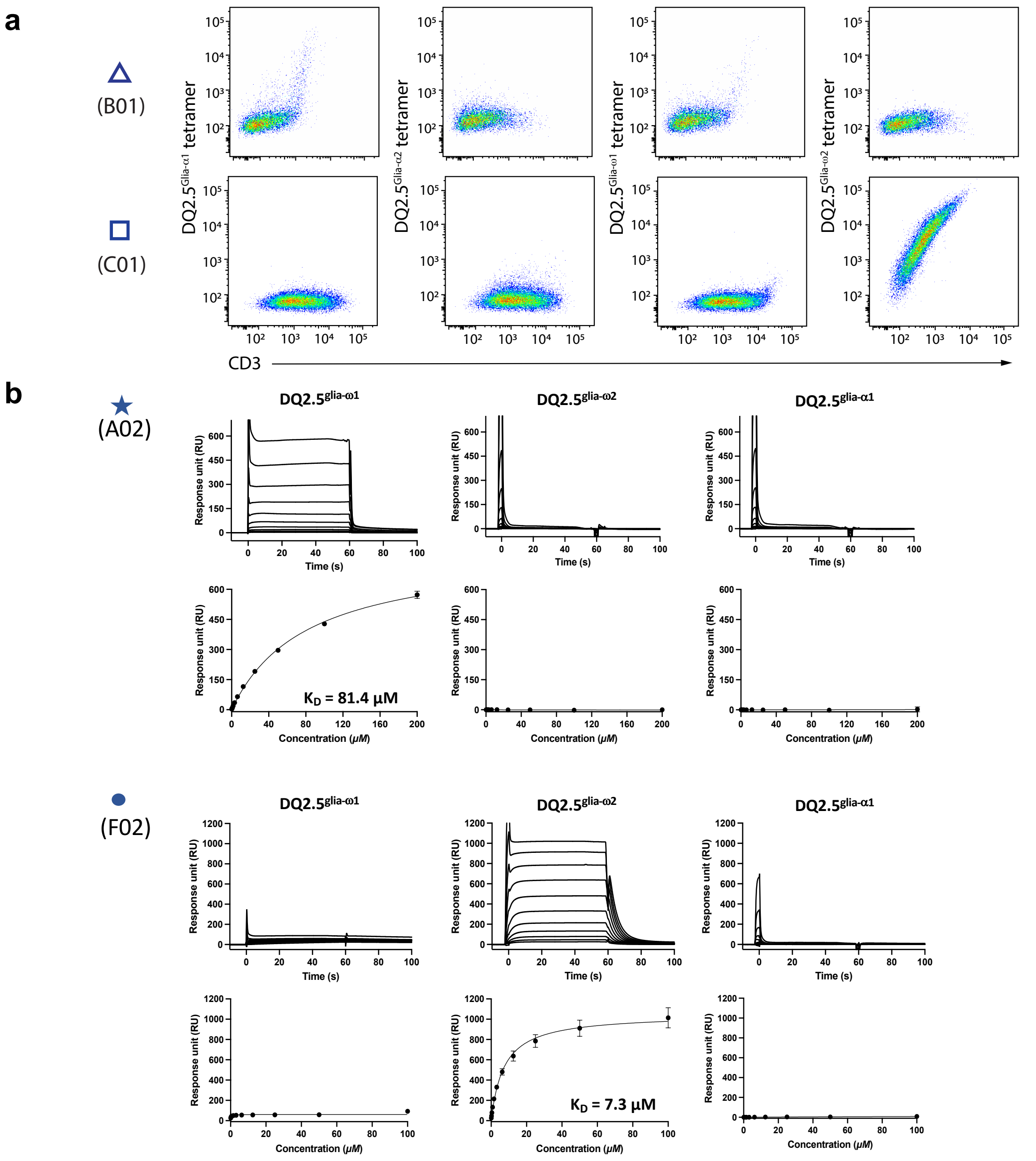

Supplementary Figure 2. Specific binding of selected T cell clone to HLA-DQ2 presenting glia peptide. a 293T cells transiently co-transfected with TCR from selected clones in Fig. 1a (identified by symbols) and CD3γδεζ were stained with either HLA-DQ2.5$^{glia\text{-}\alpha1}$ tetramer, HLA-DQ2.5$^{glia\text{-}\alpha2}$ tetramer, HLA-DQ2.5$^{glia\text{-}\omega1}$ tetramer, or HLA-DQ2.5$^{glia\text{-}\omega2}$ tetramer. **b** Affinity measurement of TCRs (selected clones in Fig. 1a (identified by symbols) against HLA-DQ2.5 $^{glia\text{-}\alpha1/glia\text{-}\omega1/glia\text{-}\omega2}$ interactions.HLA-DQ8 $^{glia\text{-}\alpha1}$ was immobilised in the reference flow cell to control non-specific binding. For K_D determination, all data were derived from two independent experiments in duplicate and curve fits using a 1:1 binding model. For each concentration, the points represent the mean and the error bars correspond to ± s.e.m.

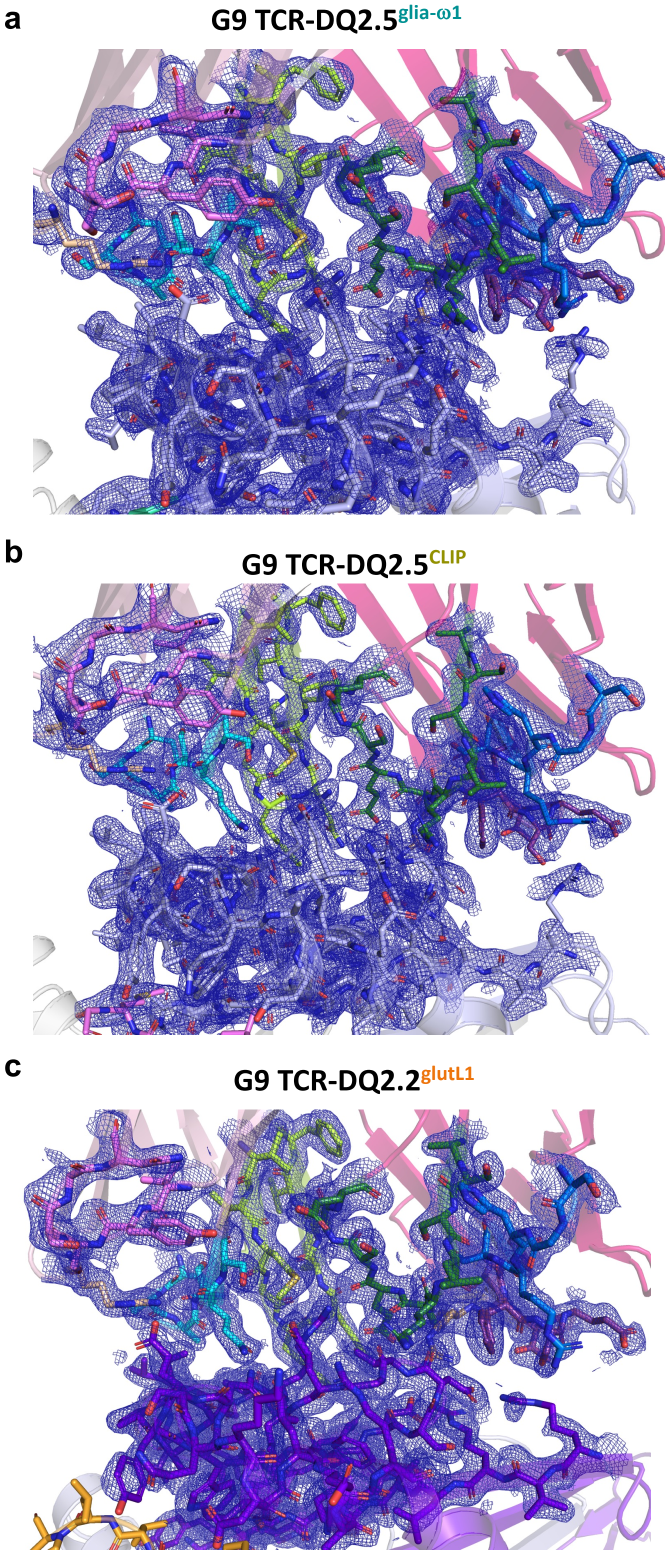

Supplementary Figure 3. Composite SA-omit σA-weighted 2Fo-Fc map for interface residues of G9-TCR in complexed with a DQ2.5^{CLIP}, b DQ2.5$^{glia\text{-}\omega 1}$, and c DQ2.2^{glutL1} contoured at 1.0σ. The composite SA-omit maps were shown in blue density mass. The HLA-DQ2.5 α- and β-chains are coloured in white and light blue, respectively, whereas the HLA-DQ2.2 α- and β-chains are coloured in white and purple, respectively. The glia-ω1, CLIP, and glutL1 peptides are coloured in greencyan, yellow, and orange sticks, respectively. The CDR loops 1α, 2α, and 3α are highlighted in cyan, violet, and light green colour, whereas 1β, 2β, 3β are coloured in blue, purple, and dark green, respectively. The FW α residues are colour in sand and β residues are colour in beige.

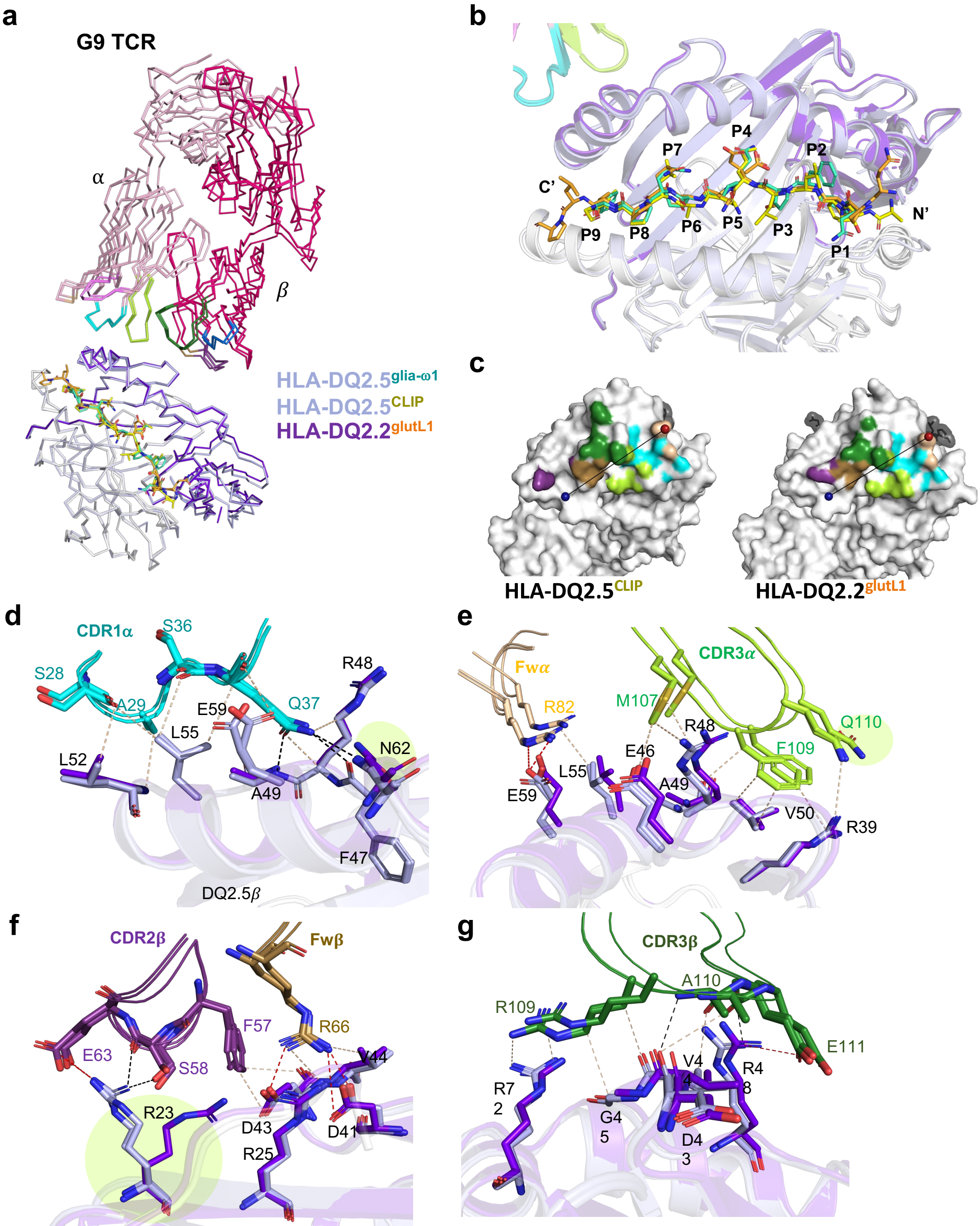

Supplementary Figure 4. Superposed structure, TCR footprint and detailed interactions of G9-TCR in complexed with DQ2.5^{CLIP}, DQ2.5$^{glia\text{-}\omega1}$ and DQ2.2^{glutL1}. **a** Overall structure superposition of G9 TCR in complexed with DQ2.5^{CLIP}, DQ2.5$^{glia\text{-}\omega1}$ and DQ2.2^{glutL1}. **b** Superposed G9 TCR-DQ2-peptide complexes at the peptide binding cleft. The HLA-DQ2.5 α- and β-chains are coloured in white and light blue, respectively, whereas the HLA-DQ2.2 α- and β-chains are coloured in white and purple, respectively. The glia-ω1, CLIP, and glutL1 peptides are coloured in greencyan, yellow, and orange sticks, respectively. The CDR loops 1α, 2α, and 3α are highlighted in cyan, violet, and light green colour, whereas 1β, 2β, 3β are coloured in blue, purple, and dark green, respectively. The FWα residues are colour in sand and β residues are colour in beige. **c** G9-TCR footprint on DQ2.5^{CLIP} and DQ2.2^{glutL1}. TCR footprint colours are in accordance with the nearest TCR contact residue. The Vα and Vβ centre of mass position are shown in red and blue spheres, respectively, and connected via a black line. Superposed interactions of G9 TCR between **a** CDR1α, **b** FWα and CDR3α, **c** CDR2β and FWβ, **d** CDR3β with HLA-DQ2.5 $^{glia\text{-}\omega1}$, DQ2.5^{CLIP} and DQ2.2^{glutL1} are shown. The deviations in interactions between DQ2.2 (light blue) and DQ2.5 (purple) were highlighted in yellow shade. All amino acids are indicated in single-letter abbreviations.

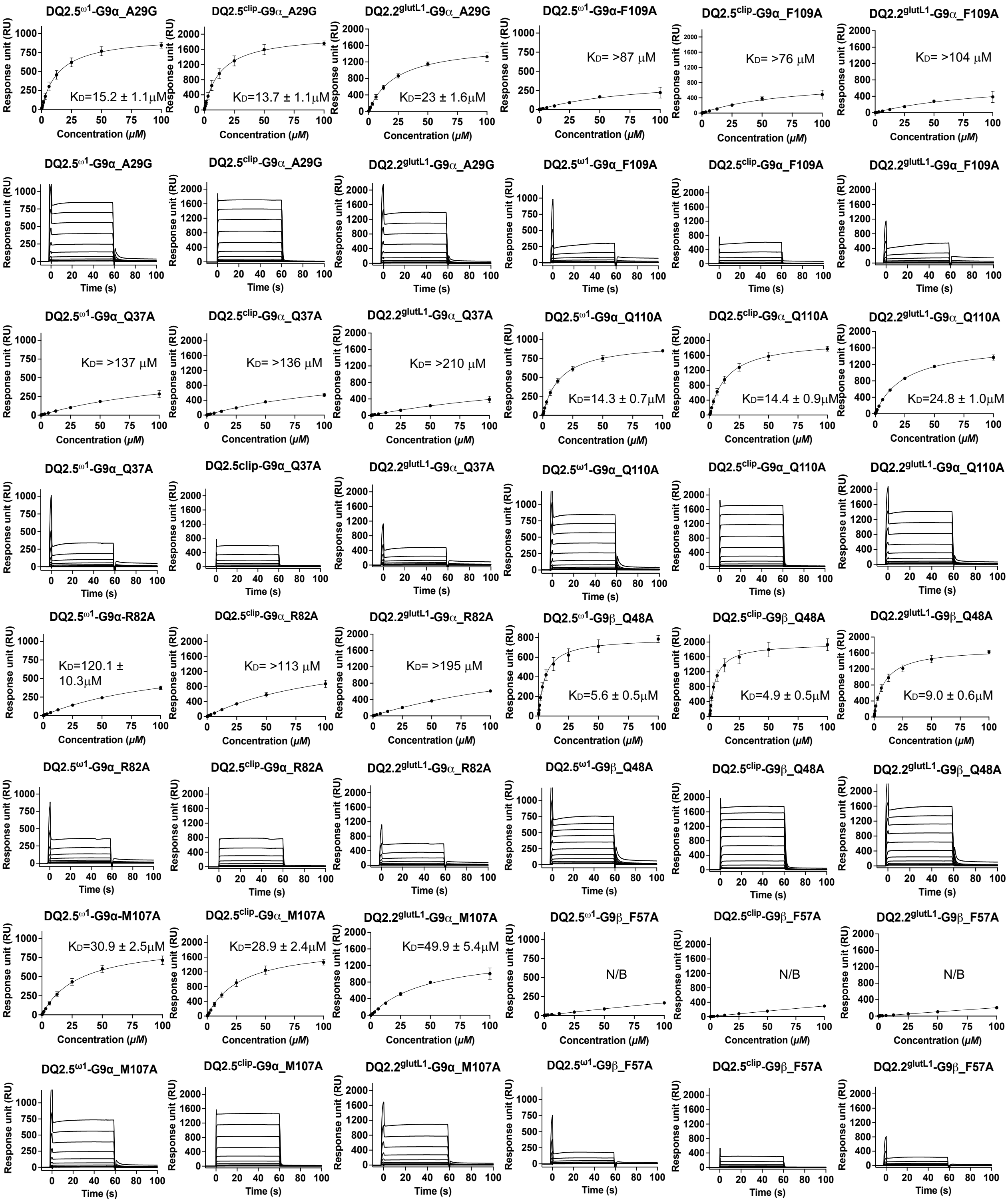

Supplementary Figure 5. Affinity analysis of point mutations on G9-TCR toward HLA-DQ2.5^{CLIP}. Binding analysis of each G9-TCR point mutation toward DQ2.5^{CLIP}, DQ2.5$^{glia-\omega1}$ and DQ2.2^{glutL1} were determined by SPR. All data derived from three independent experiments and K_D determination using a single ligand binding model. To control nonspecific binding, HLA-DQ8$^{glia-\alpha1}$ was used as reference flow cell. Equilibrium response curves were normalised against the calculated maximum response and the measurements then combined. For each concentration the points represent the mean and the error bars correspond to ± s.e.m.

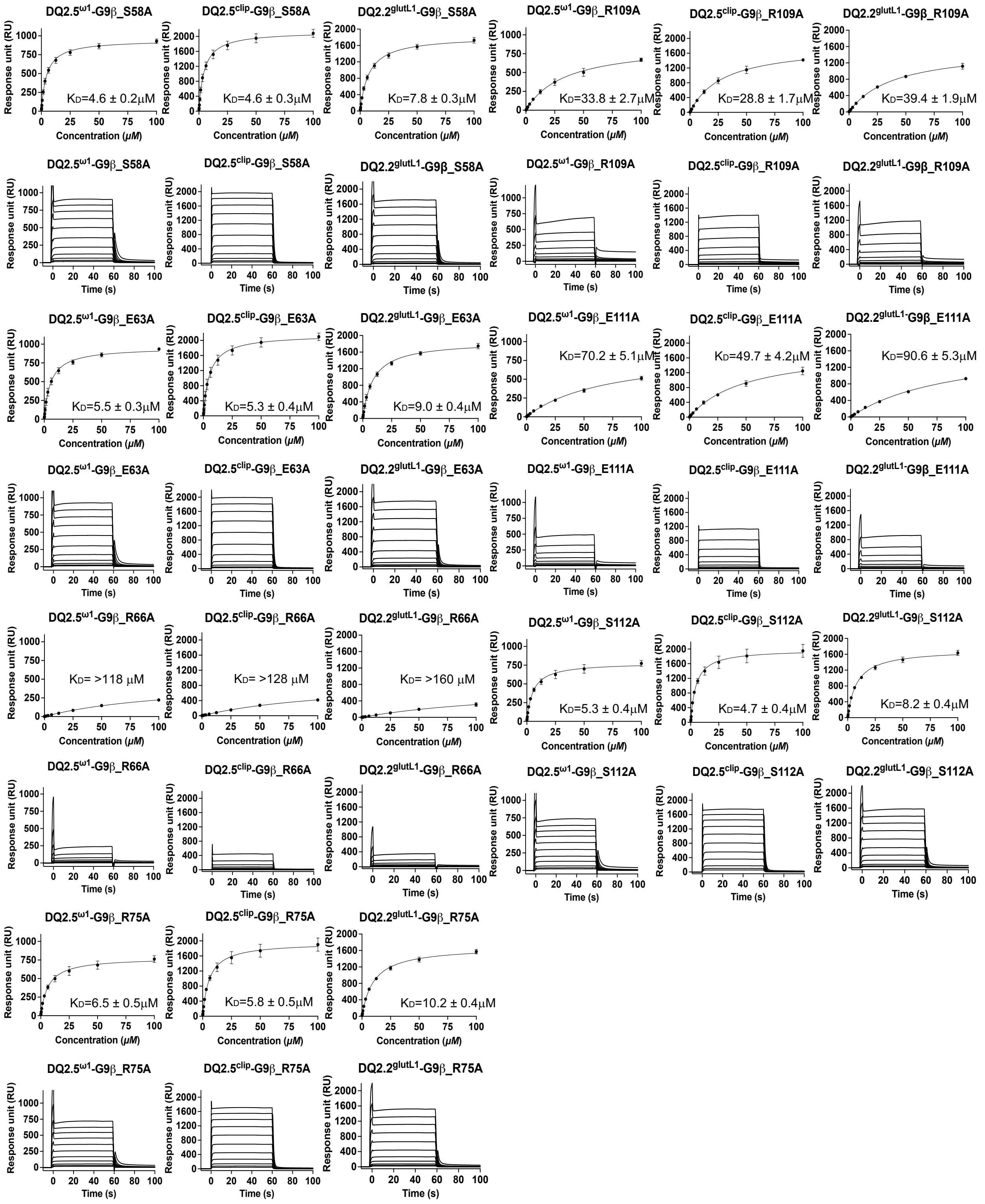

Supplementary Figure 5 continued. Affinity analysis of point mutations on G9-TCR toward HLA-DQ2.5^{CLIP}. Binding analysis of each G9-TCR point mutation toward DQ2.5^{CLIP}, DQ2.5$^{glia\text{-}\omega1}$ and DQ2.2^{glutL1} were determined by SPR. All data derived from three independent experiments and K_D determination using a single ligand binding model. To control nonspecific binding, HLA-DQ8$^{glia\text{-}\alpha1}$ was used as reference flow cell. Equilibrium response curves were normalised against the calculated maximum response and the measurements then combined. For each concentration the points represent the mean and the error bars correspond to ± s.e.m.

HLA-DR	HLA-DQ	HLA-DP	HLA-A	HLA-B	HLA-C
DRB1*01:01	DQA1*05:01, DQB1*02:01	DPA1*01:03, DPB1*01:01	A*01:01	B*07:02	B*52:01
DRB1*01:02	DQA1*03:01, DQB1*02:01	DPA1*02:01, DPB1*01:01	A*02:01	B*08:01	B*53:01
DRB1*01:03	DQA1*04:01, DQB1*02:01	DPA1*01:03, DPB1*02:01	A*02:03	B*13:01	B*54:01
DRB1*03:01	DQA1*02:01, DQB1*02:01	DPA1*02:02, DPB1*05:01	A*02:06	B*13:02	B*55:01
DRB1*03:02	DQA1*02:01, DQB1*02:02	DPA1*01:03, DPB1*03:01	A*03:01	B*14:01	B*56:01
DRB1*04:01	DQA1*02:01, DQB1*04:01	DPA1*01:05, DPB1*03:01	A*11:01	B*14:02	B*57:01
DRB1*04:02	DQA1*03:03, DQB1*04:01	DPA1*02:01, DPB1*03:01	A*11:02	B*15:01	B*57:03
DRB1*04:04	DQA1*02:01, DQB1*04:02	DPA1*01:03, DPB1*04:01	A*23:01	B*15:02	B*58:01
DRB1*04:05	DQA1*04:01, DQB1*04:02	DPA1*01:03, DPB1*04:02	A*24:02	B*15:03	B*59:01
DRB1*04:03	DQA1*01:01, DQB1*05:01	DPA1*02:01, DPB1*05:01	A*24:03	B*15:10	B*67:01
DRB1*07:01	DQA1*01:02, DQB1*05:02	DPA1*02:01, DPB1*06:01	A*25:01	B*15:11	B*73:01
DRB1*08:01	DQA1*01:03, DQB1*06:01	DPA1*01:03, DPB1*06:01	A*26:01	B*15:12	B*78:01
DRB1*09:01	DQA1*01:02, DQB1*06:02	DPA1*02:01, DPB1*09:01	A*29:01	B*15:13	B*81:01
DRB1*09:02	DQA1*01:01, DQB1*06:02	DPA1*02:02, DPB1*10:01	A*29:02	B*15:16	B*82:01
DRB1*10:01	DQA1*01:03, DQB1*06:03	DPA1*01:03, DPB1*11:01	A*30:01	B*18:01	C*01:02
DRB1*11:01	DQA1*01:02, DQB1*06:04	DPA1*01:03, DPB1*28:01	A*30:02	B*27:05	C*02:02
DRB1*11:04	DQA1*01:02, DQB1*06:09	DPA1*02:01, DPB1*13:01	A*31:01	B*27:08	C*03:02
DRB1*12:01	DQA1*03:01, DQB1*03:01	DPA1*02:02, DPB1*13:01	A*32:01	B*35:01	C*03:03
DRB1*12:02	DQA1*02:01, DQB1*03:01	DPA1*03:01, DPB1*13:01	A*33:01	B*37:01	C*03:04
DRB1*13:01	DQA1*05:03, DQB1*03:01	DPA1*02:01, DPB1*14:01	A*33:03	B*38:01	C*04:01
DRB1*13:03	DQA1*05:05, DQB1*03:19	DPA1*02:01, DPB1*15:01	A*34:01	B*39:01	C*05:01
DRB1*14:01	DQA1*06:01, DQB1*03:01	DPA1*02:01, DPB1*17:01	A*34:02	B*40:01	C*06:02
DRB1*14:02	DQA1*02:01, DQB1*03:02	DPA1*02:01, DPB1*18:01	A*36:01	B*40:02	C*07:02
DRB1*14:54	DQA1*03:01, DQB1*03:02	DPA1*01:05, DPB1*18:01	A*43:01	B*40:06	C*08:01
DRB1*15:01	DQA1*03:02, DQB1*03:02	DPA1*01:04, DPB1*18:01	A*66:01	B*41:01	C*12:03
DRB1*15:02	DQA1*02:01, DQB1*03:03	DPA1*01:03, DPB1*19:01	A*66:02	B*42:01	C*14:02
DRB1*15:03	DQA1*03:01, DQB1*03:03	DPA1*03:01, DPB1*20:01	A*68:01	B*44:02	C*15:02
DRB1*16:01	DQA1*03:02, DQB1*03:03	DPA1*01:03, DPB1*23:01	A*68:02	B*44:03	C*16:01
DRB1*16:02		DPA1*01:05, DPB1*28:01	A*69:01	B*45:01	C*17:01
DRB3*01:01		DPA1*04:01, DPB1*28:01	A*74:01	B*49:01	C*18:02
DRB3*02:02		DPA1*02:02, DPB1*11:01	A*80:01	B*47:01	
DRB3*03:01				B*48:01	
DRB4*01:01				B*46:01	
DRB4*01:03				B*50:01	
DRB5*01:01				B*51:01	
DRB5*02:02				B*51:02	

MFI

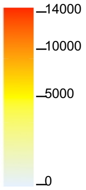

Supplementary Figure 6. Luminex immunoassay of G9 TCR for HLA class I and II detection. Heatmap representation of G9 TCR associated with HLA class II molecules (HLA-DR, -DQ, -DP), and HLA-class I molecules (HLA-A, -B, and -C). Heatmap is plotted corresponding to reactivity of G9 TCR, and the fluorescence signal (MFI) was measured as a read out (n=2). Heatmap bar is coloured from yellow to red indicates the MFI intensity.

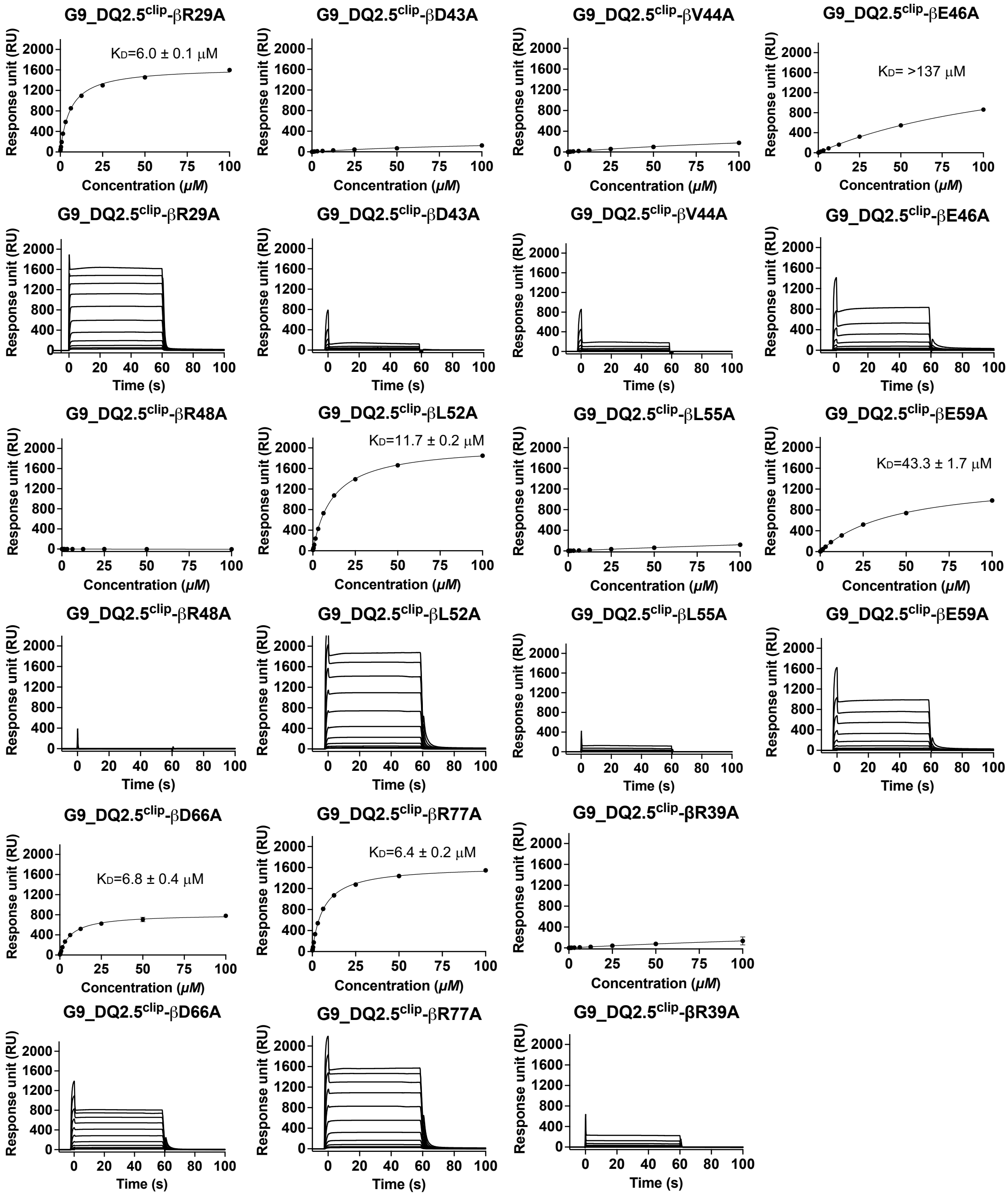

Supplementary Figure 7. Affinity analysis of G9-TCR toward point mutation of HLA-DQ2.5^{CLIP}. Binding analysis of G9-TCR toward each point mutation of DQ2.5^{CLIP} was determined by SPR. All data derived from three independent experiments and K_D determination using a single ligand binding model (n=3). For each concentration the points represent the mean and the error bars correspond to ± s.e.m. To control nonspecific binding, HLA-DQ8$^{glia\text{-}\alpha1}$ was used as reference flow cell. Equilibrium response curves were normalised against the calculated maximum response and the measurements then combined.

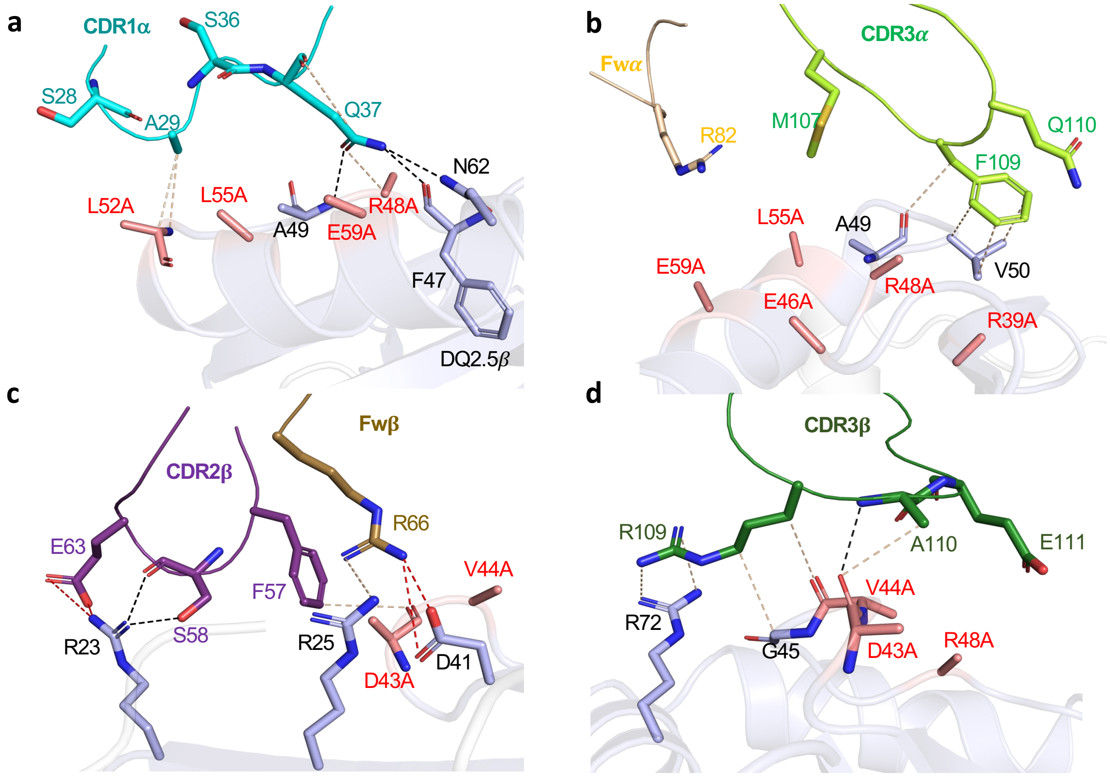

Supplementary Figure 8. Impact of alanine mutations of HLA-DQ2.5 to G9 TCR interactions (corresponded to Fig. 6a and 6b). Detailed interactions of G9 TCR between **a** CDR1α, **b** FWα and CDR3α, **c** CDR2β and FWβ, **d** CDR3β with HLA-DQ2.5$^{glia\text{-}\omega1}$ are shown. Alanine substitution residues were shown in red sticks. The CDR loops 1α, 2α, and 3α are highlighted in cyan, violet, and light green colour, whereas 1β, 2β, 3β are coloured in blue, purple, and dark green, respectively. The FWα residues are coloured in beige and FWβ residues are colour in sand. The H-bonds, VdW, and salt bridges were displayed as black, light beige, and red dash lines, respectively. All amino acids are indicated in single-letter abbreviations.

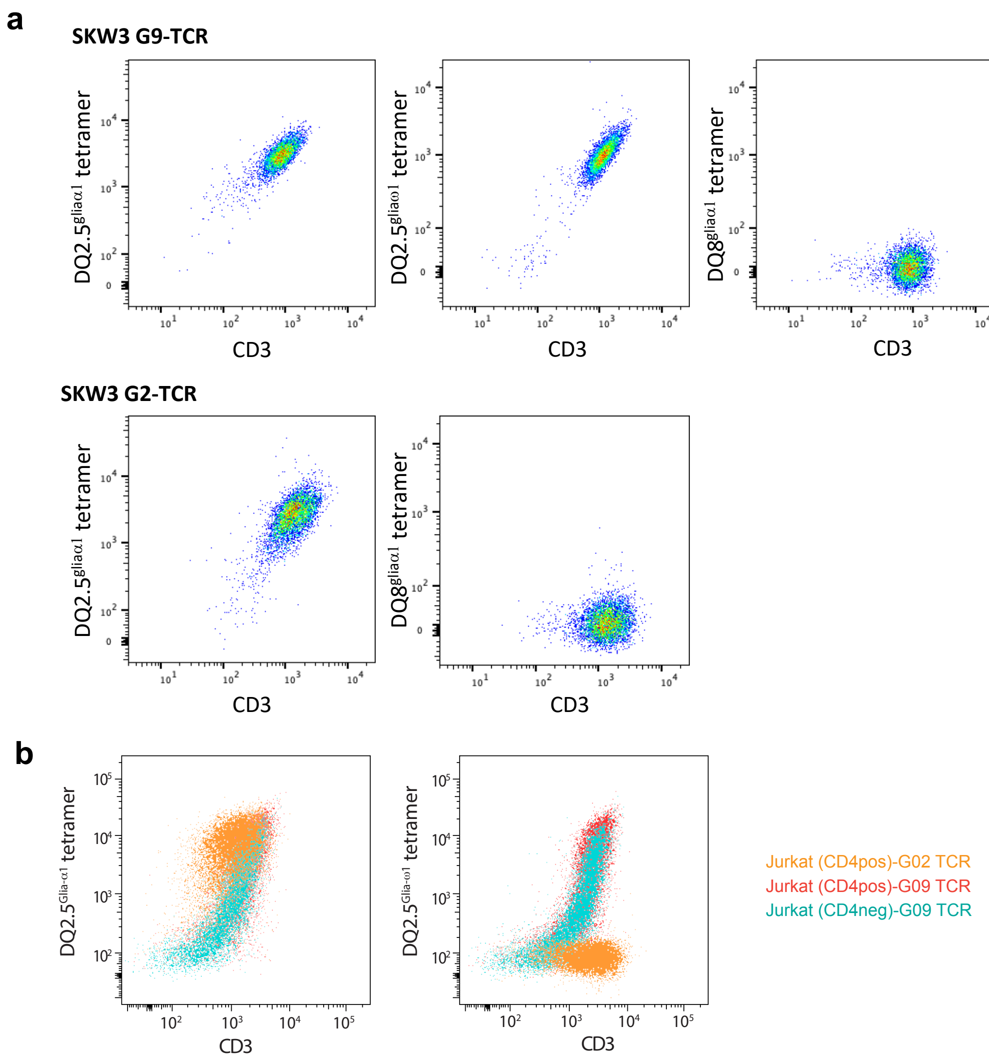

Supplementary Figure 9. Tetramer staining analyses of stably transduced G9 TCR or G2 TCR (relating to Fig. 7a & b, supplementary Fig. 1c & d). a HLA-DQ8$^{glia\text{-}\alpha1}$ tetramer staining analysis of SKW3 transduced with G9-TCR or with glia-α1/ω1 cross-reactive control G2-TCR. **b** CD4$^{+/-}$Jurkat cell lines transduced with G9-TCR or with glia-α1/ω1 cross-reactive control G2-TCR.

Supplementary Table 1. Invariant chain and gluten epitopes sequences

Peptides	Amino acid sequence	Sequence identity
DQ2.5-glia-ω1	QPFPQPEQPFP	100%
DQ2.5-glia-α1	QPFPQPELPYP	82%
DQ2.5-glia-α2	APQPELPYPQP	55%
DQ2.5-glia-ω2	APQPEQPFPWQ	73%
DQ2.5-CLIP	ATPLLMQALPMGA	27%
DQ2.2-glutL1	QPPASEQEQPVLP	36%
DQ8-glia-α1	GEGSFQPSQENP	36%

Underlined sequences denote peptide-binding register to HLA-DQ from P1-P9

Sequence identity was aligned using the DQ2.5-glia-ω1 peptide sequence as reference

Supplementary Table 2. Data collection and refinement statistics of TCR-HLA-DQ2 ternary structures

	G9 TCR-DQ2.5$^{glia\text{-}\omega1}$	G9 TCR-DQ2.5^{CLIP}	G9 TCR-DQ2.2^{glutL1}
**Data collection**			
Space group	I121	I121	I121
Cell dimensions			
a, b, c (Å)	92.95, 43.36, 257.59	92.97, 43.27, 257.96	101.84, 81.83, 143.17
α, β, γ (°)	90, 90.10, 90	90, 90.31, 90	90, 107.92, 90
Resolution (Å)[a]	45.09-2.20 (2.27-2.20)	45.22-2.45 (2.55-2.45)	48.45-2.20 (2.26-2.20)
R_{sym} or R_{merge} [a]	0.12 (1.07)	0.12 (0.62)	0.09 (0.26)
I /σ (I)[a]	9.6 (2.5)	7.1 (2.2)	9.7 (4.6)
CC1/2	0.995 (0.830)	0.988 (0.735)	0.996 (0.974)
Completeness (%)[a]	100.0 (99.8)	100.0 (100.0)	100.0 (100.0)
Redundancy [a]	5.6 (5.7)	4.4 (4.6)	5.3 (5.4)
**Refinement**			
Resolution (Å)	45.09-2.20	45.22-2.45	48.45-2.20
No. reflections	52957	38486	56912
R_{work} / R_{free}	0.195/0.225	0.176/0.232	0.185/0.220
No. atoms	6855	6638	7042
Protein	6295	6291	6452
Ligand/ion	67	50	66
Water	493	297	524
B-factors (Å^2)	39.4	41.8	34.5
Protein	39.2	41.7	33.9
Ligand/ion	48.62	64.9	54.7
Water	40.76	41.6	38.4
R.m.s. deviations			
Bond lengths (Å)	0.004	0.003	0.002
Bond angles (°)	0.716	0.579	0.569

[a] Values in parentheses refer to the highest resolution shell

Supplementary Table 3. TCR-pMHC II complex statistics

**TCR-pMHC**	**BSA** ($Å^2$)	**Vα** (%)	**Vβ** (%)	**Peptide** (%)	**1α** (%)	**2α** (%)	**3α** (%)	**Fα** (%)	**1β** (%)	**2β** (%)	**3β** (%)	**Fβ** (%)
G9 TCR-DQ2.5$^{glia\text{-}\omega1}$	1460	59.4	40.6	-	31.5	-	22.2	5.7	-	13.8	17.7	9.1
G9 TCR-DQ2.5^{clip}	1430	57.0	43.0	-	30.0	-	21.7	5.3	-	13.6	20.4	9.0
G9 TCR-DQ2.2^{glutL1}	1430	55.0	45.0	-	28.7	-	20.4	6.0	-	16.8	19.1	9.0

Supplementary Table 4. Contact table of G9 TCR-HLA-DQ2.5$^{glia\text{-}\omega1}$

TCR segment	TCR residues	HLA-DQ2.5/ω1	Type of bond
**CDR1α**	S28	L52β, L55β	VDW
	A29	L52β	VDW
	S36	L55β	VDW
	Q37(NE2) (OE1)	F47β(O), R48β, A49β(N), L55β, E59β, N62β(ND2)	HB, VDW
**FWα**	R82(NH2)	L55β, E59β(OE2)	VDW, SB
**CDR3α**	M107	E46β, R48β	VDW
	F109	R39β, A49β, V50β	VDW
	Q110	R39β	VDW
**CDR2β**	F57	D43β	VDW
	S58(OG)(O)	R23β (NE)(NH1)	HB, VDW
	E63	R23β	SB
**FWβ**	R66(NH2) (NH1)	R25β(NH2), D41β(OD2), D43β(OD2), V44β	VDW, HB, SB
**CDR3β**	R109	D43β, V44β, G45β, R72β	VDW
	A110(N)	D43β(O), V44β	VDW, HB
	E111(O)	R48β(NH1) (NH2)	VDW, HB

VDW: Van der Waals interaction (cut-off at 4 Å)
HB: hydrogen bond (cut-off at 3.5 Å)
SB: salt bridge (cut-off at 4.5 Å)

Supplementary Table 5. Contact table of G9 TCR-HLA-DQ2.5^{CLIP}

TCR segment	TCR residues	HLA-DQ2.5^{CLIP}	Type of bond
**CDR1α**	S28	L52β, L55β	VDW
	A29	L52β, L55β	VDW
	S36	L55β	VDW
	Q37(NE2) (OE1) (O)(NE2)	F47β(O), R48β, A49β(N), L55β, E59β(OE2), N62β(ND2)	HB, VDW
**FWα**	R82(NH2)	L55β, E59β(OE2)	VDW, SB
**CDR3α**	M107	R48β	VDW
	F109	R39β, A49β, V50β	VDW
**CDR2β**	F57	D43β	VDW
	S58(O)	R23β (NH2)	HB, VDW
	E63	R23β	SB
**FWβ**	R66 (NH2) (NH1)	R25β, D41β (OD2), D43β(OD2), V44β	VDW, HB, SB
**CDR3β**	R109	D43β, V44β, G45β, R72β	VDW
	A110(N)	D43β(O), V44β	VDW, HB
	E111(O)	R48β(NH1) (NH2)	VDW, HB

VDW: Van der Waals interaction (cut-off at 4 Å)
HB: hydrogen bond (cut-off at 3.5 Å)
SB: salt bridge (cut-off at 4.5 Å)

Supplementary Table 6. Contact table of G9 TCR-HLA-DQ2.2^{glutL1}

TCR segment	TCR residues	HLA-DQ2.5/glutL1	Type of bond
**CDR1α**	S28	L52β, L55β	VDW
	A29	L52β	VDW
	S36	L55β	VDW
	Q37(NE2) (OE1) (NE2)	F47β(O), R48β, A49β(N), L55β, E59β(OE2)	HB, VDW
**FWα**	R82(NH2)	L55β, E59β(OE2)	VDW, SB
**CDR3α**	M107	R48β	VDW
	F109	R39β, A49β, V50β	VDW
**CDR2β**	F57	D43β	VDW
	S58(OG)	R23β (NE)	HB, VDW
**FWβ**	R66 (NH2) (NH1)	R25β(NH2), D41β (OD2), D43β(OD2), V44β	VDW, HB, SB
**CDR3β**	R109	D43β, V44β, G45β, R72β	VDW
	A110(N)	D43β(O), V44β	VDW, HB
	E111(O)	R48β(NH1) (NH2)	VDW, HB, SB

VDW: Van der Waals interaction (cut-off at 4 Å)
HB: hydrogen bond (cut-off at 3.5 Å)
SB: salt bridge (cut-off at 4.5 Å)

Supplementary Table 7. Antibody list

Antibody name	Clone	Company	Dilution used	Catalog number
Alexa Fluor 700 Mouse anti-human CD14	M5E2	BD Biosciences	1:100	557923
Alexa Fluor 700 Mouse anti-human CD19	HIB19	BD Biosciences	1:200	557921
BV480 Mouse anti-human CD3	UCHT1	BD Biosciences	1:200	566105
BUV395 mouse anti-human CD4	SK3	BD Biosciences	1:400	563550
APC mouse anti-human CD3	UCHT1	Biolegend	1:200	300412
BUV395 mouse anti-human CD3	UCHT1	BD Biosciences	1:100	563546
APC Mouse Anti-Human CD69	FN50	BD Biosciences	1:100	555533
BD Horizon™ Fixable Viability Stain 700 (FVS700)		BD Biosciences	1:1000	564997
LIVE/DEAD™ Fixable Aqua Dead Cell Stain Kit, for 405 nm excitation		ThermoFisher Scientific	1:800	L34957
Zombie NIR™ Fixable Viability Kit		Biolegend	1:1000	423106

Supplementary Table 8. Oligonucleotides targeting human T cell receptor α (TRA) and β (TRB) genes

TRA gene(s)	External primer sequence	Internal primer sequence	Reference
TRAV1	5’ AACTGCACGTACCAGACATC 3’	5’ GCACCCACATTTCTKTCTTAC 3’	39
TRAV2	5’ GATGTGCACCAAGACTCC 3’	5’ CACTCTGTGTCCAATGCTTAC 3’	39
TRAV3	5’ AAGATCAGGTCAACGTTGC 3’	5’ ATGCACCTATTCAGTCTCTGG 3’	39
TRAV4	5’ CTCCATGGACTCATATGAAGG 3’	5’ ATTATATCACGTGGTACCAACAG 3’	39
TRAV5	5’ CTTTTCCTGAGTGTCCGAG 3’	5’ TACACAGACAGCTCCTCCAC 3’	39
TRAV6	5’ CACCCTGACCTGCAACTATAC 3’	5’ TGGTACCGACAAGATCCAG 3’	39
TRAV7	5’ GCAAAATACAGGGATGGG 3’	5’ TATGAGAAGCAGAAAGGAAGAC 3’	
TRAV8-1	5’ CTCACTGGAGTTGGGATG 3’	5’ GTCAACACCTTCAGCTTCTC 3’	39
TRAV8-2, 8-4	5’ GCCACCCTGGTTAAAGG 3’	5’ AGAGTGAAACCTCCTTCCAC 3’	39
TRAV8-3	5’ CACTGTCTCTGAAGGAGCC 3’	5’ TTTGAGGCTGAATTTAAGAGG 3’	39
TRAV8-6	5’ GAGCTGAGGTGCAACTACTC 3’	5’ AACCAAGGACTCCAGCTTC 3’	39
TRAV8-7	5’ CTAACAGAGGCCACCCAG 3’	5’ ATCAGAGGTTTTGAGGCTG 3’	39
TRAV9-1, 9-2	5’ TGGTATGTCCAATATCCTGG 3’	5’ GAAACCACTTCTTTCCACTTG 3’	39
TRAV10	5’ CAAGTGGAGCAGAGTCCTC 3’	5’ GAAAGAACTGCACTCTTCAATG 3’	39
TRAV12-1, 12-2, 12-3	5’ CARTGTTCCAGAGGGAGC 3’	5’ AAGATGGAAGGTTTACAGCAC 3’	39
TRAV13-1	5’ CATCCTTCAACCCTGAGTG 3’	5’ TCAGACAGTGCCTCAAACTAC 3’	39
TRAV13-2	5’ CAGCGCCTCAGACTACTTC 3’	5’ CAGTGAAACATCTCTCTCTGC 3’	39
TRAV14	5’ AAGATAACTCAAACCCAACCAG 3’	5’ AGGCTGTGACTCTGGACTG 3’	39
TRAV16	5’ AGTGGAGCTGAAGTGCAAC 3’	5’ GTCCAGTACTCCAGACAACG 3’	39
TRAV17	5’ GGAGAAGAGGATCCTCAGG 3’	5’ CCACCATGAACTGCAGTTAC 3’	39
TRAV18	5’ TCCAGTATCTAAACAAAGAGCC 3’	5’ TGACAGTTCCTTCCACCTG 3’	39
TRAV19	5’ AGGTAACTCAAGCGCAGAC 3’	5’ TGTGACCTTGGACTGTGTG 3’	39
TRAV20	5’ CACAGTCAGCGGTTTAAGAG 3’	5’ TCTGGTATAGGCAAGATCCTG 3’	39
TRAV21	5’ TTCCTGCAGCTCTGAGTG 3’	5’ AACTTGGTTCTCAACTGCAG 3’	39
TRAV22	5’ GTCCTCCAGACCTGATTCTC 3’	5’ CTGACTCTGTGAACAATTTGC 3’	39
TRAV23	5’ TGCTTATGAGAACACTGCG 3’	5’ TGCATTATTGATAGCCATACG 3’	39
TRAV24	5’ CTCAGTCACTGCATGTTCAG 3’	5’ TGCCTTACACTGGTACAGATG 3’	39
TRAV25	5’ GGACTTCACCACGTACTGC 3’	5’ TATAAGCAAAGGCCTGGTG 3’	39

TRAV26-1	5’ GCAAACCTGCCTTGTAATC 3’	5’ CGACAGATTCACTCCCAG 3’	39
TRAV26-2	5’ AGCCAAATTCAATGGAGAG 3’	5’ TTCACTTGCCTTGTAACCAC 3’	39
TRAV27	5’ TCAGTTTCTAAGCATCCAAGAG 3’	5’ CTCACTGTGTACTGCAACTCC 3’	39
TRAV29	5’ GCAAGTTAAGCAAAATTCACC 3’	5’ CTGCTGAAGGTCCTACATTC 3’	39
TRAV30	5’ CAACAACCAGTGCAGAGTC 3’	5’ AGAAGCATGGTGAAGCAC 3’	39
TRAV34	5’ AGAACTGGAGCAGAGTCCTC 3’	5’ ATCTCACCATAAACTGCACG 3’	39
TRAV35	5’ GGTCAACAGCTGAATCAGAG 3’	5’ ACCTGGCTATGGTACAAGC 3’	39
TRAV36	5’ GAAGACAAGGTGGTACAAAGC 3’	5’ ATCTCTGGTTGTCCACGAG 3’	39
TRAV38-1, 38-2	5’ GCACATATGACACCAGTGAG 3’	5’ CAGCAGGCAGATGATTCTC 3’	39
TRAV39	5’ CTGTTCCTGAGCATGCAG 3’	5’ TCAACCACTTCAGACAGACTG 3’	39
TRAV40	5’ GCATCTGTGACTATGAACTGC 3’	5’ GGAGGCGGAAATATTAAAGAC 3’	39
TRAV41	5’ AATGAAGTGGAGCAGAGTCC 3’	5’ TTGTTTATGCTGAGCTCAGG 3’	39
TRAC	5’ GACCAGCTTGACATCACAG 3’	5’ TGTTGCTCTTGAAGTCCATAG 3’	39

TRB gene(s)	External primer sequence	Internal primer sequence	Reference
TRBV2	5’ TCGATGATCAATTCTCAGTTG 3’	5’ TTCACTCTGAAGATCCGGTC 3’	39
TRBV3-1	5’ CAAAATACCTGGTCACACAG 3’	5’ AATCTTCACATCAATTCCCTG 3’	39
TRBV4-1, 4-2, 4-3	5’ TCGCTTCTCACCTGAATG 3’	5’ CCTGCAGCCAGAAGACTC 3’	39
TRBV5-1, 5-3, 5-4	5’ GATTCTCAGGKCKCCAGTTC 3’	5’ CTTGGAGCTGGRSGACTC 3’	39
TRBV5-5, 5-6, 5-7, 5-8	5’ GTACCAACAGGYCCTGGGT 3’	5’ TCTGAGCTGAATGTGAACG 3’	39
TRBV6-1, 6-2, 6-3, 6-5, 6-6, 6-7, 6-8, 6-9	5’ ACTCAGACCCCAAAATTCC 3’	5’ GTGTRCCCAGGATATGAACC 3’	39
TRBV6-4	5’ ACTGGCAAAGGAGAAGTCC 3’	5’ TGGTTATAGTGTCTCCAGAGC 3’	39
TRBV7-1, 7-2, 7-3	5’ TRTGATCCAATTTCAGGTCA 3’	5’ TCYACTCTGAMGWTCCAGCG 3’	39
TRBV7-4, 7-6, 7-7, 7-8, 7-9	5’ CGSWTCTYTGCAGARAGGC 3’	5’ TGRMGATYCAGCGCACA 3’	39
TRBV9	5’ GATCACAGCAACTGGACAG 3’	5’ GTACCAACAGAGCCTGGAC 3’	39
TRBV10-1	5’ CAGAGCCCAAGACACAAG 3’	5’ TGGTATCGACAAGACCTGG 3’	
TRBV10-2	5’ ACCTTGATGTGTCACCAGAC 3’	5’ GGAACACCAGTGACTCTGAG 3’	
TRBV11-1, 11-2, 11-3	5’ CGATTTTCTGCAGAGACGC 3’	5’ GACTCCACTCTCAAGATCCA 3’	39
TRBV12-3, 12-4, 12-5	5’ ARGTGACAGARATGGGACAA 3’	5’ CYACTCTGARGATCCAGCC 3’	39

TRBV13	5’ AGCGATAAAGGAAGCATCC 3’	5’ CATTCTGAACTGAACATGAGC 3’	39
TRBV14	5’ CCAACAATCGATTCTTAGCTG 3’	5’ ATTCTACTCTGAAGGTGCAGC 3’	39
TRBV15	5’ AGTGACCCTGAGTTGTTCTC 3’	5’ ATAACTTCCAATCCAGGAGG 3’	39
TRBV16	5’ GTCTTTGATGAAACAGGTATGC 3’	5’ CTGTAGCCTTGAGATCCAGG 3’	39 (External only)
TRBV17	5’ CAGACCCCCAGACACAAG 3’	5’ TGTTCACTGGTACCGACAG 3’	39
TRBV18	5’ CATAGATGAGTCAGGAATGCC 3’	5’ CGATTTTCTGCTGAATTTCC 3’	39
TRBV19	5’ AGTTGTGAACAGAATTTGAACC 3’	5’ TTCCTCTCACTGTGACATCG 3’	39
TRBV20-1	5’ AAGTTTCTCATCAACCATGC 3’	5’ ACTCTGACAGTGACCAGTGC 3’	39
TRBV23-1	5’ GCGATTCTCATCTCAATGC 3’	5’ GCAATCCTGTCCTCAGAAC 3’	39
TRBV24-1	5’ CCTACGGTTGATCTATTACTCC 3’	5’ GATGGATACAGTGTCTCTCGA 3’	39
TRBV25-1	5’ ACTACACCTCATCCACTATTCC 3’	5’ CAGAGAAGGGAGATCTTTCC 3’	39
TRBV27, 28	5’ TGGTATCGACAAGACCCAG 3’	5’ TTCYCCCTGATYCTGGAGTC 3’	39
TRBV29-1	5’ TTCTGGTACCGTCAGCAAC 3’	5’ TCTGACTGTGAGCAACATGAG 3’	39
TRBV30	5’ TCCAGCTGCTCTTCTACTCC 3’	5’ AGAATCTCTCAGCCTCCAGAC 3’	39
TRBC	5’ TAGAACTGGACTTGACAGCG 3’	5’ TTCTGATGGCTCAAACACAG 3’	39

Primers targeting TRAV and TRBV genes are sense. Primers targeting TRAC and TRBC genes are antisense. TRAV, T cell receptor Vα, TRAC, T cell receptor Cα; TRBV, T cell receptor Vβ, TRBC, T cell receptor Cβ.
