# Supplementary material for: A naturally selected αβ T cell receptor binds HLA-DQ2 molecules without co-contacting the presented peptide
Source: Nat Commun. 2025 Apr 8;16:3330. doi: 10.1038/s41467-025-58690-w (PMC11979002; doi:10.1038/s41467-025-58690-w)
Supplement: Supplementary file 2 — Reporting Summary [file 41467_2025_58690_MOESM2_ESM.pdf]

nature portfolio

Corresponding author(s): Jamie Rossjohn

Last updated by author(s): Mar 26, 2025

Reporting Summary

Nature Portfolio wishes to improve the reproducibility of the work that we publish. This form provides structure for consistency and transparency in reporting. For further information on Nature Portfolio policies, see our Editorial Policies and the Editorial Policy Checklist.

Statistics

For all statistical analyses, confirm that the following items are present in the figure legend, table legend, main text, or Methods section.

n/a	Confirmed	
☐	☒	The exact sample size (*n*) for each experimental group/condition, given as a discrete number and unit of measurement
☐	☒	A statement on whether measurements were taken from distinct samples or whether the same sample was measured repeatedly
☐	☒	The statistical test(s) used AND whether they are one- or two-sided<br>*Only common tests should be described solely by name; describe more complex techniques in the Methods section.*
☒	☐	A description of all covariates tested
☒	☐	A description of any assumptions or corrections, such as tests of normality and adjustment for multiple comparisons
☐	☒	A full description of the statistical parameters including central tendency (e.g. means) or other basic estimates (e.g. regression coefficient) AND variation (e.g. standard deviation) or associated estimates of uncertainty (e.g. confidence intervals)
☐	☒	For null hypothesis testing, the test statistic (e.g. *F*, *t*, *r*) with confidence intervals, effect sizes, degrees of freedom and *P* value noted<br>*Give P values as exact values whenever suitable.*
☒	☐	For Bayesian analysis, information on the choice of priors and Markov chain Monte Carlo settings
☒	☐	For hierarchical and complex designs, identification of the appropriate level for tests and full reporting of outcomes
☒	☐	Estimates of effect sizes (e.g. Cohen's *d*, Pearson's *r*), indicating how they were calculated

Our web collection on statistics for biologists contains articles on many of the points above.

Software and code

Policy information about availability of computer code

Data collection	FACS data: BD FACSDiva 8.0.1 software (BD Immunocytometry Systems). Surface plasmon resonance (SPR) data: Biacore T200 (Cytiva). Crystallography data: macromolecule data collection and processing implemented at the Australian Synchrotron MX2 beamline.
Data analysis	For FACS data analysis: Flowjo v10.9.0 (Three Star); For single cell TCR TRAV/TRBV usage analysis: databank search engines IMGT/V-QUEST (http://www.imgt.org/IMGT_vquest/vquest?livret=0&Option=TcR). For SPR and figure 7b generation: Prism-9 and Prism-10 (Graphpad). For crystallographic procedures: phenix-1.20.1-4487 (https://phenix-online.org), ccp4-8.0 and COOT-0.9.8.91 (https://www.ccp4.ac.uk), XDS automated data processing provided by Australian synchrotron; All structural figures were generated by PyMol v2.4.0.

For manuscripts utilizing custom algorithms or software that are central to the research but not yet described in published literature, software must be made available to editors and reviewers. We strongly encourage code deposition in a community repository (e.g. GitHub). See the Nature Portfolio guidelines for submitting code & software for further information.

Data

Policy information about availability of data

All manuscripts must include a data availability statement. This statement should provide the following information, where applicable:

- Accession codes, unique identifiers, or web links for publicly available datasets
- A description of any restrictions on data availability
- For clinical datasets or third party data, please ensure that the statement adheres to our policy

Data availability. The X-ray crystal structures were deposited in the Worldwide Protein Data Bank (https://www.rcsb.org/) with the following accession codes: G9

TCR-HLA-DQ2.5glia-w1, 9EJG[https://doi.org/10.2210/pdb9EJG/pdb]; G9 TCR-HLA-DQ2.5CLIP, 9EJH[https://doi.org/10.2210/pdb9EJH/pdb]; G9 TCR-HLA-DQ2.2glutL1, 9EJI[https://doi.org/10.2210/pdb9EJI/pdb]. All data generated in this study are provided in the Supplementary Information/Source Data File. Source data are provided as a Source Data file. Source data are provided with this paper.

Research involving human participants, their data, or biological material

Policy information about studies with human participants or human data. See also policy information about sex, gender (identity/presentation), and sexual orientation and race, ethnicity and racism.

Reporting on sex and gender	Donor 0648 is female. Sex and gender-based analyses not considered in study design
Reporting on race, ethnicity, or other socially relevant groupings	Donor 0648 is a DQA1*05:01/DQB1*02:01+ coeliac disease donor.<br>The race and ethnicity is not relevant in this study.
Population characteristics	Donor 0648, female and 54 years old; on a gluten-free diet for >6 months
Recruitment	Peripheral blood was collected into lithium heparin vacutainers (Becton Dickinson) six days after the donor undertook a 3-day gluten challenge by consuming four slices of commercial white bread daily (approximately 10g/day of wheat gluten)
Ethics oversight	Patients with coeliac disease were recruited after provision of informed consent (Human Research Ethics Committees: Royal Melbourne Hospital ID: 2020.162; The Walter and Eliza Hall Institute of Medical Research ID: 03/04). Human experimental work was conducted according to the Australian National Health and Medical Research Council (NHMRC) Code of Practice.

Note that full information on the approval of the study protocol must also be provided in the manuscript.

Field-specific reporting

Please select the one below that is the best fit for your research. If you are not sure, read the appropriate sections before making your selection.

☒ Life sciences ☐ Behavioural & social sciences ☐ Ecological, evolutionary & environmental sciences

For a reference copy of the document with all sections, see nature.com/documents/nr-reporting-summary-flat.pdf

Life sciences study design

All studies must disclose on these points even when the disclosure is negative.

Sample size	No sample size calculations were undertaken. Sample size for SPR and T cell stimulation experiments were chosen based on observed reproducibility of experimental outcomes in preliminary experiments: For SPR n≥ 2 independent experiments were performed. These sample sizes have been previously used by our laboratory: Petersen et al Nat Struct Mol Biol. 2020 Jan;27(1):49-61. doi: 10.1038/, s41594-019-0353-4; Beringer et al Nat Immunol. 2015 Nov;16(11):1153-61.doi: 10.1038/ni.3271. The sample size for PBMC (n=1) did not influence our study because our aim is to identify specific TCR toward peptide MHC, these sample sizes have been previously used by our laboratory: Lim et al., Sci. Immunol. 2021 Apr 16;6(58):eabe0896. doi: 10.1126/sciimmunol.abe0896.
Data exclusions	No data exclusion.
Replication	Each experiment was performed at least twice independently as indicated in the figure legends.
Randomization	Randomisation was not relevant to this study because it was in vitro biochemical based analysis and was not an experimental study that required allocation into groups.
Blinding	Not relevant. This was not a clinical study so no cohort comparison (i.e. different treatment groups) or similar experiment was performed.

Reporting for specific materials, systems and methods

We require information from authors about some types of materials, experimental systems and methods used in many studies. Here, indicate whether each material, system or method listed is relevant to your study. If you are not sure if a list item applies to your research, read the appropriate section before selecting a response.

Materials & experimental systems

n/a	Involved in the study	
	x	Antibodies
	x	Eukaryotic cell lines
x		Palaeontology and archaeology
x		Animals and other organisms
x		Clinical data
x		Dual use research of concern
x		Plants

Methods

n/a	Involved in the study	
x		ChIP-seq
	x	Flow cytometry
x		MRI-based neuroimaging

Antibodies

Antibodies used

BUV395 mouse anti-human CD3 (clone UCHT1, Cat # 563546, BD Biosciences); APC Mouse Anti-Human CD69 (Clone FN50, Cat # 555533, BD Biosciences); BV480 Mouse anti-human CD3 (Clone UCHT1, BD Biosciences, Cat # 566105), Alexa Fluor 700 Mouse anti-human CD14 (clone M5E2, BD Biosciences, Cat # 557923), Alexa Fluor 700 Mouse anti-human CD19 (clone: HIB19, BD Biosciences, Cat # 557921), BUV395 mouse anti-human CD4 (clone SK3, BD Biosciences, Cat # 563550), anti-human CD3-APC antibody (clone UCHT1, Biolegend Cat. 300412), anti-HLA-DQ (clone SPV-L3), LIVE/DEAD™ Fixable Aqua Dead Cell Stain Kit, for 405 nm excitation LIVE/DEAD™ (Invitrogen, ThermoFisher Scientific, Cat # L34957), Zombie NIR™ Fixable Viability Kit (Biolegend, Cat # 423106), anti-human FcR block (Miltenyi Biotech, Cat # 130-059-901), anti-PE microbeads (Miltenyi Biotech, Cat # 130-048-801), anti-APC microbeads (Miltenyi Biotech, Cat # 130-090-855), BD Horizon Fixable viability stain 700 (BD Horizon, Cat # BD564997)

Validation

BUV395 mouse anti-human CD3 (clone UCHT1, cat. no. 563546, BD Biosciences), BV480 Mouse anti-human CD3 (Clone UCHT1, BD Biosciences, Cat # 566105):
PMID: 6788570; Beverley PC, Callard RE. Distinctive functional characteristics of human "T" lymphocytes defined by E rosetting or a monoclonal anti-T cell antibody. Eur J Immunol. 1981; 11(4):329-334. (Clone-specific)
PMID: 6980937; Burns GF, Boyd AW, Beverley PC. Two monoclonal anti-human T lymphocyte antibodies have similar biologic effects and recognize the same cell surface antigen. J Immunol. 1982;129(4):1451-1457.

APC Mouse Anti-Human CD69 (Clone FN50, cat no. 555533, BD Biosciences):
Knapp W. W. Knapp .. et al., ed. Leucocyte typing IV : white cell differentiation antigens. Oxford New York: Oxford University Press; 1989:1-1182.
Schlossman SF. Stuart F. Schlossman .. et al., ed. Leucocyte typing V : white cell differentiation antigens : proceedings of the fifth international workshop and conference held in Boston, USA, 3-7 November, 1993. Oxford: Oxford University Press; 1995.

anti-HLA-DQ (clone SPV-L3):
PMID: 3279117; Velde AAT et al., Modulation of phenotypic and functional properties of human peripheral blood monocytes by IL-4. J Immunol. 1988; Mar 1;140(5):1548-54.
PMID: 6609821; Spits H et. al., ed. HLA-DC antigens can serve as recognition elements for human cytotoxic T lymphocytes. Eur J Immunol.1984; Apr;14(4):299-304

Alexa Fluor 700 Mouse anti-human CD14 (clone M5E2, BD Biosciences, Cat # 557923):
Knapp W. W. Knapp .. et al., ed. Leucocyte typing IV : white cell differentiation antigens. Oxford New York: Oxford University Press; 1989:1-1182.
PMID: 1698311; Wright SD, Ramos RA, Tobias PS, Ulevitch RJ, Mathison JC. CD14, a receptor for complexes of lipopolysaccharide (LPS) and LPS binding protein. Science. 1990; 249(4975):1431-1433.

Alexa Fluor 700 Mouse anti-human CD19 (clone: HIB19, BD Biosciences, Cat # 557921):
PMID: 7690791; Bradbury LE, Goldmacher VS, Tedder TF. The CD19 signal transduction complex of B lymphocytes. Deletion of the CD19 cytoplasmic domain alters signal transduction but not complex formation with TAPA-1 and Leu 13. J Immunol. 1993; 151 (6):2915-2927.
PMID: 2665173; Favaloro EJ, Moraitis N, Koutts J, Exner T, Bradstock KF. Endothelial cells and normal circulating haemopoietic cells share a number of surface antigens. Thromb Haemost. 1989; 61(2):217-224.

BUV395 mouse anti-human CD4 (clone SK3, BD Biosciences, Cat # 563550):
PMID: 6454755; Engleman EG, Benike CJ, Glickman E, Evans RL. Antibodies to membrane structures that distinguish suppressor/cytotoxic and helper T lymphocyte subpopulations block the mixed leukocyte reaction in man. J Exp Med. 1981; 154(1):193-198.
PMID: 6787593; Evans RL, Wall DW, Platsoucas CD, et al. Thymus-dependent membrane antigens in man: inhibition of cell-mediated lympholysis by monoclonal antibodies to TH2 antigen. Proc Natl Acad Sci U S A. 1981; 78(1):544-548. (Immunogen: Flow cytometry, Inhibition).

anti-human CD3-APC antibody (clone UCHT1, Biolegend Cat. 300412):
PMID:32719138; Crawford MP, et al. CD4 T cell-intrinsic role for the T helper 17 signature cytokine IL-17: Effector resistance to immune suppression. PNAS. 2020 Aug 11;117(32):19408-19414.
PMID: 29331015; Toepfner N et al. Detection of human disease conditions by single-cell morpho-rheological phenotyping of blood. Elife. 2018 Jan 13; 7:e29213.

LIVE/DEAD™ Fixable Aqua Dead Cell Stain Kit, for 405 nm excitation LIVE/DEAD™ (Invitrogen, ThermoFisher Scientific, Cat # L34957)

PMID:19168629; Idoyaga J, et al. Antibody to Langerin/CD207 localizes large numbers of CD8alpha+ dendritic cells to the marginal zone of mouse spleen. PNAS. 2009 Feb 3:106(5):1524-9.
PMID:19380804; George Makedonas et.al. Rapid up-regulation and granule-independent transport of perforin to the immunological synapse define a novel mechanism of antigen-specific CD8+ T cell cytotoxic activity. J Immunol. 2009 May 1;182(9):5560-9.

Zombie NIR™ Fixable Viability Kit (Biolegend, Cat # 423106):
PMID: 26982733; Headley M, et al. Visualization of immediate immune responses to pioneer metastatic cells in the lung. Nature. 2016 Mar 24;531(7595):513-7.
PMID: 32760720; Wang L, et al. SLIT2 Overexpression in Periodontitis Intensifies Inflammation and Alveolar Bone Loss, Possibly via the Activation of MAPK Pathway. Front Cell Dev Biol. 2020 Jul 14; 8:593.

anti-human FcR block (Miltenyi Biotech, Cat # 130-059-901), :
PMID: 20440073; La Gruta NL et al. Primary CTL response magnitude in mice is determined by the extent of naive T cell recruitment and subsequent clonal expansion. J Clin Invest. 2010: 120, 1885-1894.
PMID: 17707129; Moon JJ, et al. Naive CD4(+) T cell frequency varies for different epitopes and predicts repertoire diversity and response magnitude. Immunity. 2007: 27, 203-213.

anti-PE microbeads (Miltenyi Biotech, Cat # 130-048-801), anti-APC microbeads (Miltenyi Biotech, Cat # 130-090-855):
PMID: 15162426; Barnes, E. et al. Ultra-sensitive class I tetramer analysis reveals previously undetectable populations of antiviral CD8 + T cells. Eur J Immunol. 2004 Jun;34(6): 1570-7.
PMID: 15194806; Lucas, M. et al. Ex vivo phenotype and frequency of influenza virus-specific CD4 memory T cells. J Virol. 2004 Jul; 78 (13): 7284-7.

BBD Horizon Fixable viability stain 700 (BD Horizon, Cat # BD564997):
PMID: 19135024; Barny Abrams et al. 3-Carboxy-6-chloro-7-hydroxycoumarin: ahighly fluorescent, water-soluble violet-excitable dye for cell analysis. Anal Biochem. 2009 March 15; 386(2):262-9.
PMID: 18178815; Yvonne Burmeister et al. ICOS controls the pool size of effector-memory and regulatory T cells. J Immunol.2008 Jan15;180 (2):774-82

Eukaryotic cell lines

Policy information about cell lines and Sex and Gender in Research

Cell line source(s)	HEK293T(ATCC, #CRL-3216), jurkat cells, and Raji B cells were from ATCC. SKW3 line (ACC 53) was sourced from German Collection of Microorganisms and Cell Cultures (DSMZ). High Five Cells (BTI-TN-5B1-4) were from Thermo Fisher Scientific.
Authentication	HLA-DQ expression was confirmed on Raji cells by staining with anti-DQ monoclonal antibody and FACS analyses. Absence of CD3 expression was confirmed on SKW3 parental cells by staining with anti-CD3 monoclonal antibody and subsequent FACS analyses. T cell receptor (TCR) transduction of SKW3/jurkat cells were confirmed by CD3 (indicating TCR surface expression). HEK293T from ATCC and High Five Cells (BTI-TN-5B1-4) from Thermo Fisher Scientific were not authenticated.
Mycoplasma contamination	Absence of mycoplasma contamination in cell lines was confirmed via PCR.
Commonly misidentified lines (See ICLAC register)	No commonly misidentified cell lines were used in the study.

Plants

Seed stocks	N/A
Novel plant genotypes	not relevant in this study
Authentication	not relevant in this study

Flow Cytometry

Plots

Confirm that:

- [x] The axis labels state the marker and fluorochrome used (e.g. CD4-FITC).
- [x] The axis scales are clearly visible. Include numbers along axes only for bottom left plot of group (a 'group' is an analysis of identical markers).
- [x] All plots are contour plots with outliers or pseudocolor plots.
- [x] A numerical value for number of cells or percentage (with statistics) is provided.

Methodology

Sample preparation	HLA-DQ2.5-glia-w1-PE and HLA-DQ2.5-glia-w2-APC restricted CD4+ T cells sorted from human PBMC: cryopreserved PBMC were thawed and rested overnight at 37°C, 5% CO2. Cells were counted and 13.6 million PBMC were treated with 50 nM dasatinib for 30 min at 37 °C, then stained with PE- or APC- labelled tetramers (at 10 µg/ml final concentration) for 1 h at room temperature. Cells were then washed and labelled with anti-PE or anti-APC conjugated magnetic microbeads, and tetramer-bound cells were enriched over a magnetic LS column (Miltenyi Biotec). Enriched cells were then stained with a cocktail of conjugated antibodies to identify epitope-specific cells from naïve CD4+ T cell populations (CD14, CD19, CD3, CD4, and FVS700 or Live/Dead Fixable Near-IR viability stain).<br>For T cell stimulation assay and In vitro TCR expression : Cells were harvested by centrifugation and resuspended/washed with Phosphate buffered saline (PBS). PBS containing 2% fetal bovine serum was used for antibody and or HLA-DQ tetramer staining.
Instrument	FACSAria III cell sorter, BD LSRFortessa™ X-20
Software	FlowJo v10
Cell population abundance	For single-cell sorting samples a 'post-sort' flow cytometry analysis was not possible.
Gating strategy	Tetramer-binding CD4+ cells for single-cell sorting were gated as follows: single cells (FSC-A/FSC-H), lymphocytes (FSC-A/SSC-A), Live, Dump negative cells (FVS700 negative, CD14-AF700 and CD19-AF700 negative), human CD3+ (CD3-BV480 positive), human CD4+ (CD4-BUV395 positive). See Supplementary FIgure 1A.<br><br>T cell stimulation assay – TCR transduced SKW3/jurkat cells were gated as follows: cells (FCS-A/SSC-H); single cells (FSC-A/FSC-H); live cells (Zombie NIR viability stain, RB780-A low); G9-SKW3/jurkat: human CD4 (CD4-V450); human CD3 (CD3-BUV-395) versus human CD69 (APC-CD69) was analysed to observed the CD3 down regulation and CD69 up-regulation upon T cell activation.<br><br>In vitro TCR expression using HEK293T cells were gated as follows: Cells (FCS-A/SSC-H); single cells (FSC-A/FSC-H); live cells (Aqua Blue viability stain, BV525-A low); GFP high (eGFP B530); Tetramer (PE YG585) versus mouse TCR (TCR-APC or TCR-APC-Cy7) or human CD3 (CD3-BUV395).

- [x] Tick this box to confirm that a figure exemplifying the gating strategy is provided in the Supplementary Information.
